# Using Co-design to Explore How Midwives Can Support the Emerging Mother-Infant Relationship During the Early Postnatal Period: Protocol for a Mixed Methods Study

**DOI:** 10.2196/29770

**Published:** 2021-06-10

**Authors:** Cathy Stoodley, Lois McKellar, Tahereh Ziaian, Mary Steen, Ian Gwilt, Jenny Fereday

**Affiliations:** 1 UniSA Clinical & Health Sciences City East Campus University of South Australia Adelaide Australia; 2 UniSA: Justice & Society Magill Campus University of South Australia Adelaide Australia; 3 UniSA: Creative City West Campus University of South Australia Adelaide Australia; 4 Women's and Children's Health Network Adelaide Australia

**Keywords:** mother-infant relationship, mother-infant relations, mother-infant bonding, infant development, midwife, early postnatal, co-design, mixed methods

## Abstract

**Background:**

The postnatal period can be a challenging time for women, with mothers experiencing a range of emotions. As a woman transitions to motherhood, she adjusts to a new sense of self and forms a new relationship with her infant. Becoming a mother is a complex cognitive and social process that is unique for each woman and is influenced and shaped by culture. The emerging mother-infant relationship is a significant factor in maternal well-being and infant development, with the bond between the mother and her baby being critical to the development of secure attachment. It has been recognized that the strength of this relationship is the main predictor of how well a child will do throughout life. There has been a global focus on the importance of the first 1000 days, with Australia identifying this as a national priority. Midwives are ideally placed to support mothers during the development of the mother-infant relationship, providing care through the early postnatal period, which has been identified as a *sensitive period* for the development of the mother-infant relationship.

**Objective:**

The aim of this study is to explore how midwives can support the emerging mother-infant relationship in the context of cultural diversity and develop an appropriate co-designed intervention in the early postnatal period.

**Methods:**

This study will use a mixed method approach, specifically the exploratory sequential design (intervention development variant). This study will be undertaken in 3 phases: 1 qualitative phase, which is followed by 2 quantitative phases. Phase 1 will include a scoping review to explore interventions that have influenced the development of the mother-infant relationship, and then, interviews will be undertaken with women exploring their early experiences of motherhood, followed by 3 co-design workshops. The workshops will engage with multilevel stakeholder representatives where, through partnership and participation, they will propose and develop an intervention to support the emerging mother-infant relationship. Phase 2 will develop and pilot 2 purpose-designed evaluation surveys to evaluate the co-designed intervention from the perspective of both mothers and midwives. Phase 3 will implement and evaluate the co-designed intervention using pre- and postmeasures and feedback from the purpose-designed surveys.

**Results:**

Phase 1 has commenced and is expected to be completed by August 2021. Phase 2 is expected to be completed by September 2021, with phase 3 commencing in October 2021. The study will be completed by March 2023.

**Conclusions:**

The results of this study will be shared with a variety of audiences and will contribute to the body of knowledge on the mother-infant relationship, potentially improving the understanding of this relationship for women and midwives. This may result in improved strategies for care, with mothers benefiting from enhanced experience and satisfaction during the early postnatal period.

## Introduction

### Background

The mother-infant relationship is both complex and dynamic and benefits both the mother and infant. This relationship is developed through an interactional partnership, whereby the mother and her infant engage in a range of physical, cognitive, social, and affective behaviors [1]. Early interactions between the mother and her baby are essential for the formation of the mother-infant relationship, with the quality of the relationship being crucial to the psychological health and mental well-being development of the infant [2,3]. This relationship is commonly described in terms of bonding [3] and attachment [4,5].

### Maternal-Infant Bonding and Attachment

Maternal-infant attachment involves the reciprocal connection between the mother and her baby, which is developed over time [5,6], whereas maternal-infant bonding involves a one-way connection between the mother and her infant [5,7]. This bond develops progressively, initially during pregnancy, then up to and including the infant’s first year of life [5,7,8], with these first experiences forming the foundation for future relationships across a lifetime [8,9]. One of the critical periods influencing maternal-infant bonding is childbirth, where in the first few hours, maternal hormones play a positive role in enhancing sensitivity, reactivity, and receptiveness to their newborn [7]. The early bond developed between the mother and her baby is an important precursor for the development of secure attachment [6,10].

Attachment is the reciprocal relationship between the mother and her infant and can have an enormous impact on the infant’s future mental, physical, social, and emotional health. Specifically, it has been asserted that the strength of this relationship is the main predictor of how well a child will do in school and throughout life [7,11]. Secure attachment is important, as it promotes the optimal development of the baby’s nervous system, which is responsible for positive social and emotional development. It also establishes a safe base from which the child can explore and interact with the world [11,12]. Insecure attachment fails to meet the child’s need for security and understanding, disrupting the infant’s developing brain. This insecurity can inhibit emotional, mental, and physical development across the life span, leading to long-term physical and mental health challenges [11,12]. Secure attachment grows out of the success of a nonverbal communication process between the mother and baby [12,13]. Specifically, maternal-infant synchrony is a crucial aspect of the development of secure maternal-infant attachment [14]. During pregnancy, a psychosocial transition begins and continues after birth, where the woman moves from her known, current reality to an unknown, new reality in getting to know her infant [15]. Notably, due to hormones, neurochemistry, and experiences, there are dynamic changes that occur after birth, which increase the plasticity of the maternal brain, which, in turn, promotes caregiving behavior from the mother to her infant [16].

Several characteristics can influence maternal-infant synchrony, including maternal sensitivity, responsiveness, and emotional state as well as the infant’s well-being and temperament [14]. Maternal sensitivity is recognized as one of the most significant contributors to maternal-infant synchrony and is therefore essential to the development of secure attachment in the mother-infant relationship [7,17]. Maternal-infant synchrony, where there is appropriate maternal sensitivity and responsiveness, promotes infant comfort and reduces distress and disengagement. Importantly, this synchrony underpins the development of secure attachment between the mother and infant and the ongoing emotional and cognitive development of the infant [14,17]. Protecting and nurturing these aspects within the emerging mother-infant relationship is crucial.

Although many attachment theories have been based on Western populations, there is a consensus that attachment is universal and not specific to Western cultures [18]. All infants require a primary caregiver to provide warm, responsive, protective, and linguistically rich environments for appropriate growth and development, regardless of culture. These characteristics are crucial in caregiving behaviors, although the way in which this is provided may vary according to cultural norms [1]. A recognition that cultural nuances may influence parents’ responsiveness to their infant must be considered in offering support to the emerging mother-infant relationship; therefore, this study will seek to consider cultural diversity. According to the Australian Institute of Health and Welfare [19], 298,630 women gave birth in Australia in 2018, with one-quarter (27%) of these mothers born in a non-English speaking country. It has been reported by Hennegan et al [20] that culturally and linguistically diverse (CALD) women are more likely to face barriers throughout their maternity care, including challenges of relocation, distance from family and support networks, a language barrier, and potentially discriminatory or culturally insensitive care from maternity service providers.

### Role of the Midwife

The foundations of social-emotional development of the infant are based on the primary caregiving relationship. Midwives are uniquely placed to support the emerging mother-infant relationship during this childbirth continuum, as their scope of practice begins in early pregnancy and concludes at 6 weeks postnatal [21]. Midwifery care is centered around the woman and her family, where the midwife works in partnership with the woman, promoting a healthy pregnancy, physiological birth, and a positive transition to motherhood [22]. The childbirth experience can have significant implications for early bonding between the mother and infant [23].

In 1991, the World Health Organization and United Nations Children’s Fund launched the Baby-Friendly Hospital Initiative (now known as the Baby-Friendly Health Initiative), which significantly changed practices in birthing suites by implementing skin to skin to protect, promote, and support breastfeeding. Although the focus of this initiative was primarily to support exclusive breastfeeding, it was later recognized that this practice also supported the emerging mother-infant relationship [4,24]. Within the Baby-Friendly Health Initiative, there are several examples of midwives being well placed during the early postnatal period to influence the emerging mother-infant relationship. This includes the practice of skin to skin and rooming-in [24].

Kennell and Klaus [25] conceptualized the early postnatal phase as the *sensitive period*, which they defined as the first few hours or days after birth, and concluded that purposefully supporting positive mother-infant contact during this time could enhance the emerging relationship. Although more recent research has redefined the sensitive period to include pregnancy, it has also extended this time beyond the immediate postpartum period, continuing over the first few weeks and months of life [5]. Midwives provide care during the significant time when the woman transitions from pregnancy to the postnatal period and the change from her *inside* baby to *outside baby* occurs [26].

## Methods

### Study Design

The mixed methods typology chosen for this study is the exploratory sequential design, specifically the intervention development variant. This design involves 3 distinct phases: 1 qualitative phase, which is followed by 2 quantitative phases [27]. The rationale for using this approach is that the initial qualitative phase provides important insight into the phenomenon, which is vital to the development of the intervention, whereas the final quantitative phase provides an opportunity for an evaluation of the intervention [27].

### Aim and Objectives

The aim of this study is to explore how midwives can support the emerging mother-infant relationship in the context of cultural diversity and develop an appropriate co-design intervention in the early postnatal period.

The objectives of this study are as follows:

1. To undertake a scoping review to explore interventions in pregnancy and the early postnatal period to support the mother-infant relationship
2. To engage in co-design with multilevel stakeholder representatives to explore culturally diverse ways to support the emerging mother-infant relationship and to develop a co-design intervention
3. To develop and pilot 2 purpose-designed surveys to evaluate the co-designed intervention from the perspective of mothers and midwives
4. To implement and evaluate the co-designed intervention.

### Phase 1

#### Overview

Phase 1 will draw on a co-design process to explore and develop a co-designed intervention to support the emerging mother-infant relationship. Before the co-design workshops, a scoping review will be undertaken to examine the existing literature on interventions during pregnancy and/or the early postnatal period that support the mother-infant relationship. Interviews with mothers during the early postnatal period will also be undertaken to explore their experience of getting to know their baby. The findings from the scoping review and interviews will contribute to the co-design workshops and will help to inform the development of an intervention.

#### Scoping Review

A scoping review is one method that is used to review the literature, which can provide valuable information to the investigator when undertaking research [28,29]. This scoping review will explore interventions in pregnancy and the early postnatal period to support the mother-infant relationship. The review of the literature will seek to answer the following questions:

- What types of interventions have been used to support the mother-infant relationship from pregnancy to 6 weeks?
- What format was used to deliver the interventions?
- What interventions conducted in Western and non-Western countries have considered cultural diversity?
- What interventions have been evaluated?

A protocol for the scoping review will be developed following the process outlined in the *JBI Manual for Evidence Synthesis* [30], which will be made publicly available.

#### Interviews With Mothers

Interviews with mothers will seek to understand their experiences in the early postnatal period and their perception of influences on the emerging mother-infant relationship. Gathering these data is important for strengthening the voice of mothers in the co-design process. Mothers in a postnatal ward who meet the inclusion criteria will be invited to participate and complete a short demographic survey. Mothers who give consent will then receive an activity pack, which has a series of prompts designed to encourage them to reflect on their early mother-infant experiences. These prompts will include a polaroid camera and a journal. The mothers will be asked to capture some of their early experiences by taking daily photos and describing what helped them to get to know and respond to their baby during the first 2 weeks after birth. Using photo-elicitation is a powerful tool in research for the purpose of data generation, capturing the lived experiences of participants [31]. Shohel [32] suggested using photographs as a basis for discussion during research activities instead of other methods because of the former being a less threatening and more interesting approach to promote participation and engagement. In addition, photographs can act as a *memory anchor*, assisting participants to reflect and better explain their perceptions and experiences [33].

A semistructured face-to-face or virtual interview will be conducted between 4 and 6 weeks postnatal, where the mothers can discuss their visual journey and reflections. The interview will be guided by a few open-ended questions. These questions will explore what mothers found helpful or unhelpful in getting to know their baby to gain an understanding of the emerging mother-infant relationship. Collecting and analyzing the interview data relating to the mother’s experience will provide a generative tool in the ideation process and inform the co-design. Using generative tools is helpful to aid engagement in the co-design process, as this approach promotes collaboration and imagination and encourages personal and emotional reflection [34].

#### Co-design

Co-design involves a group of people *using* creativity and collaboration during the design development process [35]. A co-design approach draws on the knowledge and expertise of both consumers and service users and clinicians and explores the care practices. The aim of using a co-design process in the research is to bring a group of multilevel stakeholder representatives together (consumers, clinicians, and professionals), who share an interest in the *study* but may have different motivations and experiences to discuss the topic, learn together, and make decisions [35]. Co-design workshops *use* a variety of activities to explore the phenomenon of interest. These workshops are helpful when considering a new project or if you want to understand the consumer’s experience and find a solution for an existing challenge [36]. The co-design workshops will provide a forum to engage in creative group activities with all participants. As the co-design process involves a diverse group of people with differences in knowledge, interests, roles, and experience, strategies will be *used* to address the power differential to ensure that equal participation occurs and all views can be expressed [37,38].

In this proposed study, the co-design workshops will bring together multilevel representatives, including mothers and professionals who contribute to supporting the early development of the mother-infant relationship. In this study, professionals will include midwives, perinatal infant mental health (PIMH) professionals, occupational therapists (OTs), and cross-cultural consultants. The rationale for including these professionals is provided below.

Midwives provide maternity care to women from early pregnancy to 6 weeks postnatal, with women highly valuing the professional support they receive from midwives [21,39]. The midwife is ideally placed during their transition to motherhood to support the mother-infant relationship, focusing on the psychological and physical changes that occur during this time [39,40].

The field of PIMH is focused on providing mental health and well-being support to women, infants, and their families during the perinatal period [41]. PIMH services support the development of early parent-infant relationships to improve mother-infant outcomes, whereby the promotion of a positive parent-infant relationship and the mother’s and baby’s mental health are key elements [42,43].

Although relatively new, OTs provide support to mothers as they transition into motherhood, recognizing the adjustment to their new occupational role as a primary caregiver. OTs provide a valuable perspective with a focus on occupational performance in early motherhood, with co-occupation significantly influencing maternal and infant health outcomes [44,45]. This can provide valuable insight into the changing life roles, routines, and coping strategies through the transition to motherhood [44,45].

In addition, cross-cultural consultants will be invited to participate to ensure culturally appropriate interactions. The role of a cross-cultural consultant involves facilitating and shaping cross-cultural and intercultural interactions, ensuring cultural influence on how problems, opportunities, and responses are viewed [46]. Cross-cultural consultants facilitate collaboration or mediation between people or groups of different cultures to produce change or resolve conflicts [47]. There are a wide variety of settings that have used cross-cultural consultants [46], with the concept largely applied to anthropology until the 1960s, where it has since emerged in health care practice [48]. The use of cross-cultural consultants in maternity care has been adopted as a strategy to support culturally appropriate care [49]. Cross-cultural consultants are vital when working with people from various cultural backgrounds, as they are familiar with the language, cultural norms, practices, and worldview. This can provide a platform for effective dialog, avoiding misunderstandings by connecting one group to another, by providing explanations and appropriate translations that enable communication and effective relationship building [46]. Cross-cultural consultants seek to build bridges between the parties to support interactions to promote respect for cultural differences [48]. Using cross-cultural consultants in health care has become a positive strategy among the immigrant population to promote equity in service provision [48].

#### Participants and Setting

Multilevel stakeholder representatives (mothers, midwives, PIMH professionals, OTs, and cross-cultural consultants) who meet the inclusion criteria (Table 1) will be invited to participate in the study using nonprobability purposive sampling at the study site hospital in South Australia.

**Table 1:** Phase 1 and 2 inclusion and exclusion criteria.

Participants	Inclusion	Exclusion
Mothers	Primiparous woman Aged ≥18 years ANRQ^a^<23 and EPDS^b^<13 (completed at first antenatal visit) Baby direct room in Term labor and birth^c^	Multiparous woman Aged <18 years Inability to give informed consent ANRQ>23 and EPDS>13 Preterm labor and/or birth Complications that may influence bonding Baby is an inpatient in a special care baby unit or neonatal intensive care unit Any previous mental illness history
Midwife	All midwives employed at the study site hospital in South Australia on the postnatal ward, home visiting, and midwifery group practice	All midwives who are not employed at the study site hospital in South Australia
Other stakeholders	PIMH^d^ professionals, occupational therapist, and cross-cultural consultant who work with mothers and their babies within the study site hospital in South Australia	Health professionals who do not work with mothers and their babies within the study site hospital in South Australia

^a^ANRQ: Antenatal Risk Questionnaire; Antenatal Risk Questionnaire is a self-report 12-item psychosocial assessment screening tool administered during the antenatal period, which aids in the prediction of women who may develop postnatal depression. A score of 23 or more may suggest the presence of significant psychosocial risk factors that require consideration in mental health care planning [50].

^b^EPDS: Edinburgh Postnatal Depression Scale; the Edinburgh Postnatal Depression Scale is a self-report 10-item measure designed to screen women for symptoms of emotional distress during pregnancy and the postnatal period, reflecting the woman’s experience in the last 7 days. A score of 13 or more and/or a positive answer to question 10 also needs to be considered as potentially clinically significant; therefore, mental health care planning should be considered [50,51].

^c^The word *birth* is inclusive of normal vaginal birth, instrumental (ventouse or forceps), and cesarean section (emergency or elective).

^d^PIMH: perinatal infant mental health.

An estimated sample size of 10-15 mothers will be recruited to participate in an interview. This estimated number is generally acceptable for interview data, recognizing that data saturation, where no new themes are identified, is generally achieved at this point [52-54]. However, due to a focus on cultural diversity, the researcher may recruit mothers until diversity is represented. To ensure that adequate cultural representation occurs, this study will aim to recruit one-third of the sample from CALD backgrounds. Representation will be sorted from 3 migration regions: Africa, South Asia, and Middle East [55]. If the participant woman with English as a second language requests an interpreter, an officially accredited interpreter from the Translating and Interpreting Service will be used via the telephone to meet the language needs of the participant. Alternatively, if the participant has a bilingual support person with them and requests that they interpret for them, then this will be acceptable. The primary researcher will use either the officially accredited interpreter or the bilingual support person. In addition, mothers will be asked if they would like to participate in the co-design workshop group.

A total of 8 to 10 multilevel stakeholder representatives who work with mothers and their babies within the study site hospital in South Australia will be invited to participate in the co-design group. This includes midwives who work in the postnatal ward, midwifery group practice, and home visits; OTs; PIMH professionals; and cross-cultural consultants.

#### Data Collection and Analysis

##### Interviews With Mothers

A short demographic survey will describe the characteristics of the participant mothers, for example, age, ethnicity, occupation, and education level. The demographic characteristics collected from the survey will be reported using descriptive statistics.

The interviews will be conducted face-to-face or by Zoom using a semistructured approach to ensure that the research aim and objectives are met while also allowing for flexibility to elaborate on any answers or to seek further clarification [53]. The journal and photos will not be analyzed; however, these will act as prompts for reflection during the interview. The interviews will be audio-recorded and transcribed verbatim to preserve participants’ experiences by using their own words without interpretation [53,56]. The data will be analyzed using thematic analysis, as described using the 6 steps by Braun et al [56]: reading—familiarization of the data, generating codes, searching for themes, reviewing themes, defining and naming themes—and writing—finalizing analysis. The data will be read and reread by the researcher to identify codes and themes; a selection of data will be reviewed by 2 supervisors. The themes will then be reviewed and checked by the researcher’s principal supervisor to ensure that all respondents’ experiences were represented in the data [56].

##### Co-design Group Workshop

Co-design workshops will be undertaken using a variety of techniques. During the first 2 workshops, which will incorporate the predesign and design development process, a variety of tools and techniques will be used, such as probes, generative toolkits, and prototypes. These techniques contribute to disarming the power imbalance and support empowerment and democratic participation [37,57]. Using probes during the predesign stage is useful, as it invites individuals to reflect on and express their feelings, responses, reactions, experiences, and attitudes. Probes are tangible artifacts that take a variety of forms, such as diaries, workbooks, cameras with instructions, and games [37,57]. This may include some of the mothers’ artifacts from the interviews (with permission).

Generative toolkits help groups communicate and collaborate to jointly create new ideas and concepts. The toolkit approach is used to facilitate collaborative activities that purposefully steer participation, reflection, bringing vision, ideas, and concepts [37,57]. Toolkits may contain a combination of 2D or 3D components, pictures, words, phrases, blocks, shapes, buttons, pipe cleaners, and wires. These activities will lead to the development of a prototype that can then be evaluated in practice [37,57]. Prototypes can take the form of physical manifestations of ideas or concepts, ranging from an idea to a finished product. Prototypes can also include a wide variety of materials, such as clay, foam, wood, plastic, and digital or electronic elements [37].

The co-design workshop analysis will occur concurrently through group feedback, collation of the discussion, and reporting findings back to the group at the end of each session until an intervention is agreed upon.

### Phase 2

#### Overview

Phase 2 will develop and pilot 2 evaluation surveys that will be used in phase 3. Two distinct purpose-designed surveys will be developed by the researchers to evaluate satisfaction and experience with the co-designed intervention for midwives and mothers: (1) midwives will complete a purpose-designed survey providing feedback on their experience of implementing the co-designed intervention and (2) mothers will complete a different survey designed to collect feedback on their experience and acceptability of the intervention.

#### Participants and Setting

The participants for phase 2 will be recruited from the co-design workshop group and will be invited to review the survey for content validity and to determine reliability. The inclusion and exclusion criteria will remain the same as for phase 1 (Table 1).

#### Data Collection and Analysis

The purpose-design surveys will consist of closed, open-ended, and Likert scale questions. Content validity will be undertaken, whereby a panel of experts will be invited to review the questionnaire and suggest ways to improve the design. The quality of the design was measured by the level of agreement between experts [58]. The recommended number of experts ranges from 2 to 9, with the content validity index used for the analysis [59]. To assess reliability, a pilot survey will be provided to the participants from the co-design group to ensure that the survey can be understood and works effectively. The test-retest method will be used to measure internal consistency, whereby the survey will be administered at different time intervals to the same participants [58,60]. In a test for agreement between 2 raters using the κ statistical test, a sample size of 14 subjects achieves 82% power to detect a true κ value of 0.70 in a test of H0: κ=ĸ0 versus H1: κ≠ĸ0 when there are 2 categories with frequencies equal to 0.50 and 0.50. This power calculation is based on a significance level of .05 [61]. Both midwives and mothers will be engaged in the pilot process (n=14). The data from the test-retest will be collated into the SPSS and analyzed, and the findings will be presented as descriptive statistics [58,62].

### Phase 3

Phase 3 will implement and evaluate the co-designed intervention as a pilot study to assess feasibility using pre-and postmeasures and acceptably using evaluation survey feedback. Education and training, as needed, will be provided to the midwives who will be offering the co-designed intervention to the mothers.

#### Pre- and Postmeasures

Pre-and postmeasure data will be collected from participant mothers to determine the effect of the co-designed intervention. Two validated self-reported scales have been selected: the Postnatal Bonding Questionnaire (PBQ) [63,64] and the Maternal Infant Responsiveness Instrument (MIRI) [65]. These tools will provide different but complementary data, measuring maternal bonding and maternal responsiveness, and will seek to establish whether the co-designed intervention influences maternal-infant bonding and responsiveness.

Brockington et al [63,64] developed the PBQ to identify relationship disturbances with the mother and infant during the postnatal period. This data collection tool is a self-rated questionnaire and has been validated in at least six studies [9]. The scale has 25 statements, each followed by 6 alternative responses ranging from *always* to *never*. Positive responses, such as “my baby is the most beautiful baby in the world,” are scored from zero (*always*) to 5 (*never*). Negative responses, such as “I am afraid of my baby,” are scored from 5 (*always*) to zero (*never*). This scale has high convergent validity with other measures of similar constructs [9]. Most studies have been undertaken after 6 weeks postpartum, with a few studies undertaken in the early postnatal period [66,67]. Alterations to factor one, specifically the question concerning smiling and playing, will need to be removed or adjusted to accommodate the early postnatal timeframe [67].

Amankwaa and Pickler [65] developed the MIRI, which is a 22-item scale designed to measure mothers’ feelings about the infant and an appraisal of the infant’s responses. Specifically, this tool measures the mother’s recognition of their responses, the mother’s recognition of the infant’s responses to them, and any difficulties they notice in responsiveness. Response options ranged from 1 (strongly agree) to 5 (strongly disagree). The internal consistency of the measure is high (α=.86) [65]. The MIRI has been used in studies of mothers of term [68] and preterm infants [69]. The 22-item self-reported MIRI is a useful tool to use in clinical research to measure parenting interventions, particularly pre-and postmeasures that assess new programs or interventions [68].

#### Participants and Setting

Using purposive sampling, mothers (n=75) in the postnatal ward at the study site hospital in South Australia will be invited to participate in the study. As this is a pilot trial, using a formal power calculation is usually not required, although it is still necessary to justify choosing a particular sample size [70]. Sample size has been determined based on advice from a statistician and guidance from the study by Whitehead et al [70] on pilot projects. The sample size seeks to detect a medium effect size of 0.5 (Cohen *d*) with a significance level of .05. This phase will draw on the same recruitment approach from phase 1, with the aim of recruiting one-third of the sample from CALD backgrounds. Representation will be sorted from the 3 migration regions (Africa, South Asia, and Middle East) [55]. If a participant woman with English as a second language requires an interpreter service, then it will be offered.

All midwives working in the postnatal ward, home visits, or midwifery group practice at the study site hospital in South Australia will be invited to participate (Textbox 1), with the aim of recruiting a minimum of 10 midwives.

**Phase 3 inclusion and exclusion criteria.**

**Inclusion Criteria**

- Mothers
  Primiparous woman
  Aged ≥18 years
  Antenatal Risk Questionnaire (ANRQ)<23 and Edinburgh Postnatal Depression Scale (EPDS)<13 (completed at first antenatal visit)
  Baby direct room in
  Term labor and birth
- Midwife
  All midwives employed at the study site hospital in South Australia on the postnatal ward, home visiting, and midwifery group practice

**Exclusion Criteria**

- Mothers
  Multiparous woman
  Aged <18 years
  Inability to give informed consent
  ANRQ>23 and EPDS>13
  Preterm labor and/or birth
  Complications that may influence bonding
  Baby is an inpatient in special care baby unit or neonatal intensive care unit
  Any previous mental illness history
- Midwife
  All midwives who are not employed at the study site hospital in South Australia

#### Data Collection and Analysis

In phase 3, the participants will be invited to complete the PBQ and MIRI before implementing the co-designed intervention (pretest). The PBQ and MIRI will be repeated after the co-designed intervention (posttest) [71]. The PBQ and MIRI will be completed by each mother on the same day. The amount of time between the pre- and posttest will be determined depending on the type of intervention developed. All participants will be matched to their pre- and postresponses as well as to their survey by allocating each participant a confidential code at the commencement of the study. The data will be analyzed using SPSS V.27 and presented as simple descriptive statistics. Paired *t* test will be used to demonstrate a change in maternal-infant bonding and responsiveness, as this test is designed to assess differences at 2 separate time points between the one group [62].

In addition, mothers and midwives who engage in the co-designed intervention will be provided with the purpose-designed surveys to evaluate satisfaction and experience with the intervention. The data will be analyzed using SPSS V.27 and presented as simple descriptive statistics. Simple descriptive statistics will describe, organize, and summarize the raw data, providing meaning through numerical data [62]. Open-ended questions will be analyzed using thematic analysis [56].

### Ethical Considerations

This study was approved by the University of South Australia Human Research Ethics Committee (application ID: 203776). In addition, the Human Research Ethics Application Women’s and Children’s Health Network approved the study on February 11, 2021 (application: 2020/HRE01717; HREC/20/WCHN/154).

## Results

Phase 1 commenced in February 2021, initially undertaking a scoping review. The recruitment and data collection for the interviews will commence in June 2021 and expected completion of the analysis is by July 2021, with the results to inform the co-design workshops in August 2021. Phase 2 is expected to be completed by September 2021, with phase 3 commencing in October 2021 and data collection and analysis to be completed by January 2022. The study will be completed by March 2023.

## Discussion

### Study Rationale

Research into the mother-infant relationship has included both normative and at-risk situations, with much of the research aimed at addressing and promoting parent-infant bonding for at-risk groups [72]. More recently, research has identified and confirmed a range of factors, including low education or income, psychosocial or depressive risk, infants with colic, infants with or at risk of developing a sleeping problem, and parents with an insensitive parenting style [73]. The literature highlights that there is a need for more research, which considers that mothers who do not meet the established *high-risk* criteria yet may be at risk and could benefit from supportive strategies during the early postnatal period. For instance, the increased prevalence of maternal anxiety and postnatal depression (1 in 5 women in Australia) may impact the mother-infant relationship [74].

In response to the impact that a disrupted mother-infant relationship can have on the infant’s well-being, a number of early parenting interventions have been developed [73]. In a meta-analysis on early parenting interventions, Mihelic et al [73] identified that many interventions had taken an educational approach, which involved teaching specific strategies and/or provision of information on infant development and behavior. These sessions were often conducted after 3 months of age and were conducted over numerous sessions and in a range of settings [73]. Given the implications for lifelong health outcomes, supporting the developing relationship during the postnatal period is vital, with midwives being ideally placed during this sensitive period to offer professional support. There is emerging research where midwives have provided support to the early relationship, for example, providing instructions during antenatal classes on singing lullabies to improve bonding [72].

Using a co-design approach whereby multilevel stakeholder representatives share their knowledge and expertise to explore care practices has become a popular technique to address consumer needs [35,36]. This study will use an inclusionary approach to engage women from CALD backgrounds to ensure adequate representation of the community when designing an inclusive intervention. This is essential considering that one-quarter (27%) of the mothers who gave birth in 2018 were born in a non–English-speaking country [19]. This study aims to scope the literature and devise a co-design intervention that can be applied during routine care for all women inclusive of culture.

### Conclusions

Research into the mother-infant relationship has predominately focused on at-risk situations. The results from this proposed study will contribute to the body of knowledge of the mother-infant relationship, in particular, mothers who are not identified but may be at risk of developing a bonding disorder. Furthermore, this study will contribute to the growing body of evidence using co-design to enhance health care practices. This research may also provide a better understanding of the mother-infant relationship for women and midwives, which could result in improved strategies for care, where mothers may benefit from an enhanced postnatal experience. The findings and results of this study will be shared with a variety of audiences, which will include the scientific community, government or policy makers, service planners, industry stakeholders or service professionals and participants and communities, and the wider community using a variety of techniques.

## Appendix

Multimedia Appendix 1 Peer review 1 from the University of South Australia.

Multimedia Appendix 2 Peer review 2 from the University of South Australia.

## Abbreviations

CALD: culturally and linguistically diverse
MIRI: Maternal Infant Responsiveness Instrument
OT: occupational therapist
PBQ: Postnatal Bonding Questionnaire
PIMH: perinatal infant mental health
