# Supplementary material for: Using Co-design to Explore How Midwives Can Support the Emerging Mother-Infant Relationship During the Early Postnatal Period: Protocol for a Mixed Methods Study
Source: JMIR Res Protoc. 2021 Jun 10;10(6):e29770. doi: 10.2196/29770 (PMC8241434; doi:10.2196/29770)
Supplement: Multimedia Appendix 1 [file resprot_v10i6e29770_app1.pdf]

University of South Australia

Confirmation of Candidature

Panel Assessment Form

**Name of Candidate**	**Cathy Stoodley**
**Name of Panel Member**	**Julie Fleet**

My opinion of the students' proposal is summarised below *[Tick where appropriate]*

**Topic**	**Satisfactory**	**Unsatisfactory**
The topic contributes new knowledge to the subject area	✓	
The topic has sufficient scope for doctoral research	✓	
The proposed research has a sound policy, philosophical, scientific or theoretical base	✓	
The proposed application of the research to issues in health and health care is discussed	✓	
To my knowledge, the material in this proposed research has not been published before	✓	

**Literature Review**	**Satisfactory**	**Unsatisfactory**
Succinct summary of the relevant literature was provided in the proposal	Some additional detail on how interventions on other cohorts have been delivered and evaluated would help to further inform rationale and methodology	
The literature cited by the student is based chiefly on primary sources	✓	
The sources of evidence on which the proposed research is based are clearly stated	✓	
The interpretations and conclusions are justified by the evidence presented	✓	

**Proposal**	**Satisfactory**	**Unsatisfactory**
The title of the research accurately reflects the content of the proposal	✓ consider including the term cultural diversity	
Clear and logical description of proposed research is presented	✓	

The writing style is grammatically correct and references are cited appropriately and correspond accurately to the conventions used by the School	✓	
A 300 word abstract is included in the proposal that accurately reflects the proposed research	✓	376 words
The research proposal does not exceed 20 pages	✓	21 pages excluding title, content page & references
Sufficient detail of proposed research is provided to allow evaluation	✓	

**Research Method**	**Satisfactory**	**Unsatisfactory**
There is a clear statement of the purpose, aim, question or hypothesis of the research	✓	
All key concepts are clearly defined (as appropriate)	✓	
Research participants are clearly described. *The description covers (as appropriate) the inclusion criteria, identification and recruitment of participants, and justification of sample size*	✓ some additional detail on recruitment is needed	
Data collection process is clearly described. *The description covers (as appropriate) what data will be collected, how the data will be collected and the processes that will be used to ensure accuracy of data. Proposal includes any data collection tools, scales or instruments that will be used*	✓	
The proposed data analysis is clearly described, justified and appropriate	✓	
The ethical aspects of the research are addressed (as appropriate)	✓	
The ethical approval and any other permissions that are required to conduct research are described	✓	
Any resource implications of the proposed research are adequately addressed	✓	
Timeframe of the proposed research is described and is appropriate	✓	
Trial Table of Contents is appropriate	✓	

Are there any cost implications for this proposed study? Yes ☒ No ☐

If yes, have these costs been addressed in the proposal? Yes ☒ No ☐

Reviewer feedback to candidate

Please include additional typed information, comments and critique of the proposal that can be sent to the candidate and supervisors

Thank you for the opportunity to review your proposal. It is an important piece of research that identifies a gap in the literature for this critical period that is often overlooked and under resourced.

I have made a few comments/suggestions that you may like to consider in track changes in the body of the document.

The abstract is well written I only suggest reference is made to the focus on cultural diversity as this is a key aim of the study that addresses one of the gaps in the literature.

The background provides a very comprehensive overview of the topic and clearly states the gaps in knowledge and the importance of supporting the mother-infant relationship. You acknowledge there are a number of interventions that target parental responsiveness, and some may argue a family centred approach would be an effective strategy to support woman-infant relationship at this time. Therefore, I've recommended you include some additional detail to clarify why you have focussed the intervention on the mother and midwife. I understand you will be conducting a scoping review in Phase 1 but could provide some detail in your background based on the 36 interventions you make reference to in the meta-analysis from Mihelic, Morawska & Filus 2017. This would help to provide detail on the type of intervention that may be considered or adapted, such as the methods that have already been trialled - how they were delivered, timeframe of the intervention, resources and how they were evaluated. This may further support your justification for your chosen topic and methodology.

You have described your chosen methods well and demonstrated a good understanding of the methodological approach. I have made comments to suggest areas that would be good to clarified further such as recruitment strategies and how each phase informs the next. For example, I was unclear as to how each stage of Phase 1 informed the next - some additional clarification is needed – will the scoping review, interviews and workshops occur concurrently as mentioned or sequentially? You may also consider adding some additional detail re the inclusion criteria for women re language considerations and age.

Overall the proposal provides a clear and logical description of your proposed research. I wish you well in progressing with this important piece of work.

Reviewer signature: ________________________________ Date: __29/9/20______________
