# Supplementary material for: Using Co-design to Explore How Midwives Can Support the Emerging Mother-Infant Relationship During the Early Postnatal Period: Protocol for a Mixed Methods Study
Source: JMIR Res Protoc. 2021 Jun 10;10(6):e29770. doi: 10.2196/29770 (PMC8241434; doi:10.2196/29770)
Supplement: Multimedia Appendix 2 [file resprot_v10i6e29770_app2.pdf]

Confirmation of Candidature

Panel Assessment Form

**Name of Candidate**	**Cathy Stoodley**
**Name of Panel Member**	**Yvonne Hauck**

My opinion of the students' proposal is summarised below *[Tick where appropriate]*

**Topic**	**Satisfactory**	**Unsatisfactory**
The topic contributes new knowledge to the subject area	√	
The topic has sufficient scope for doctoral research	√	
The proposed research has a sound policy, philosophical, scientific or theoretical base	√	
The proposed application of the research to issues in health and health care is discussed	√	
To my knowledge, the material in this proposed research has not been published before	√	

**Literature Review**	**Satisfactory**	**Unsatisfactory**
Succinct summary of the relevant literature was provided in the proposal	√	
The literature cited by the student is based chiefly on primary sources	√	
The sources of evidence on which the proposed research is based are clearly stated	√	
The interpretations and conclusions are justified by the evidence presented	√	

**Proposal**	**Satisfactory**	**Unsatisfactory**
The title of the research accurately reflects the content of the proposal	√	
Clear and logical description of proposed research is presented	√	
The writing style is grammatically correct and references are cited appropriately and correspond accurately to the conventions used by the School	√	
A 300 word abstract is included in the proposal that accurately reflects the proposed research		√
The research proposal does not exceed 20 pages	√	
Sufficient detail of proposed research is provided to allow evaluation	√	

Research Method	Satisfactory	Unsatisfactory
There is a clear statement of the purpose, aim, question or hypothesis of the research	Please refer to comments below	
All key concepts are clearly defined (as appropriate)	Please refer to comments below	
Research participants are clearly described. *The description covers (as appropriate) the inclusion criteria, identification and recruitment of participants, and justification of sample size*	Please refer to comments below	
Data collection process is clearly described. *The description covers (as appropriate) what data will be collected, how the data will be collected and the processes that will be used to ensure accuracy of data. Proposal includes any data collection tools, scales or instruments that will be used*	Please refer to comments below	
The proposed data analysis is clearly described, justified and appropriate	Please refer to comments below	
The ethical aspects of the research are addressed (as appropriate)	√	
The ethical approval and any other permissions that are required to conduct research are described	√	
Any resource implications of the proposed research are adequately addressed	√	
Timeframe of the proposed research is described and is appropriate	√	
Trial Table of Contents is appropriate	√	

Reviewer feedback to candidate

Thank you for the opportunity to review this well written proposal that addresses an important topic for all maternity health professionals but especially midwives who are ideally placed to be able to implement an intervention that may foster the mother-infant relationship in the early postnatal period. Focusing upon women with cultural diversity is also well supported in the literature and is timely and essential in multi-cultural Australia.

If word limits are not an issue, consider using 'a three phase exploratory sequential design' in the title as it is more informative.

The **proposal abstract** is 376 words although the assessment criteria suggests that it should be 300 words. Although the abstract exceeds the word guidelines it is comprehensive and highlights for the reader a synopsis for why this topic is important, the overall aim and that an exploratory sequential design will involve three phases. One query is 'development of appropriate intervention(s)….' Is the intention to develop more than one intervention? If there is potential for more than one, how will phase 2 and 3 progress? Will one intervention be selected over others and if so, what process will be undertaken to select the one intervention? I would suggest asserting that one intervention will be developed, implemented and evaluated. To address the word limit requirement, perhaps the Introduction and Background sections could be reduced and the Methods expanded in the abstract.

The **Background** (review of the literature) is comprehensive and clearly justifies the importance of promoting bonding and attachment to enhance the mother-infant relationship. Justification of using midwives due to their key role in intrapartum and early postpartum care to implement a potential intervention during this sensitive period is also well supported in the literature review. However, there is no referenced literature around other stakeholder representatives (i.e. cultural consultants, occupational therapists and perinatal mental health professionals) in the background so why they would be included in objective #2 is unclear as the focus of the study is with low risk women from diverse cultural backgrounds. Why would there be involvement of occupational therapists and mental health clinicians with low risk women? Finally, what is the role of a cultural consultant in clinical practice? Is this a translator if the woman is not comfortable with English?

Based upon the conclusion to the background literature, it appears that the intervention may cover a 6 week period post birth. Not all midwives have access to women for this period of time, especially those working in private hospitals and/or public hospitals where there may not be continuity of care models (i.e. midwifery group practices). Therefore will you be focusing upon women who are in a continuity of care model like an MGP with a known midwife who will provide follow up care to 6 weeks post birth? Midwives working only on a postnatal ward will not have experience or opportunity to follow women for 6 weeks. What role do cultural consultants, OTs and perinatal mental health professionals have working with low risk women on a postnatal ward?

Further information and clarification is required in the **methods** section. **Figure 1** should note the actual research designs being employed with each phase and not use terms like qualitative and quantitative.

As this study will involve three phases, it may be useful for the reader if the author can acknowledge which objective will be addressed in each phase. This can easily be noted in brackets after each individual objective. **Objective #2** the focus of the background was on the role of midwifery support for low risk women from culturally diverse background so why are multilevel stakeholder representatives being included? See earlier comment about justifying inclusion of these representatives for an intervention targeting midwives. **Objective #3** – why are two purpose designed survey proposed? What outcome measures will the surveys be focused upon (i.e. maternal postnatal attachment?) A maternal postnatal attachment scale does exist (Condon & Corkindale, 1998) and is available through Flinders University. Perhaps this scale could be reviewed and modified? **Objective #4** – how will the proposed intervention be evaluated (pilot RCT) and then evaluated for what outcome measures (i.e. the impact of the intervention on maternal postnatal attachment?). Consider including the outcome measures in the objective. Not until page 19 did it become apparent that postnatal bonding and maternal infant responsiveness will be the outcome measures for phase 3.

The rationale for using an exploratory sequential design is sound. The scoping review and a qualitative component will be implemented in **phase 1**, however co-design is not a research design? Please clarify what co-design refers to in the context of this study? It sounds like a philosophical framework rather than a research design or data collection technique. How will the workshops be conducted? What qualitative data will be collected (i.e. video recording, interviews, observation?). What qualitative design is being used for the interviews with the mothers (i.e. qualitative descriptive, participatory action research, ethnography)? “Affinity diagramming” isn’t clearly explained – what does the data look like and what steps are used to analyse this data? Will the workshop data and the interview data be analysed together? What ‘guided interview questions’ will be used with the mothers at 4 to 6 weeks post birth? How the workshop will be managed (i.e. activities to be used)? Will the mothers who are interviewed be the same mothers that attend the workshops? When will the workshops be scheduled (i.e. before or after women’s interviews)? Will mothers, midwives and other health professionals all attend the same workshop or will mothers be separate to ensure they feel heard, non-intimated by the presence of health professionals and willing to share their experiences?

During **phase 1,** who will approach and recruit the mothers on the postnatal ward? It is assumed that the researcher will not be involved in providing clinical care and will be unknown to the women so please confirm? Who and how will informed consent be collected from the participants? Do the mothers have to be able to speak and read English? Do the mothers have to be born outside of Australia? This isn’t specified in your inclusion/exclusion criteria? Aside from a ‘term labour and birth’ does the type of birth matter - do you want women with a vaginal birth (spontaneous or instrumental verses a planned or emergency caesarean birth)? Any particular cultural groups being targeted? “Recruitment will continue until diversity is represented” – please clarify.

Under **phase 2** in the methods, it appears that the outcomes to be measured are satisfaction of the midwives and mothers’ acceptability of the intervention. When and how will the midwives be offered training/education on how to implement the intervention? From the background literature, one can assume the intervention may include recognised factors such as skin to skin contact, supporting breastfeeding, encouraging face to face interaction with non-verbal communication such as eye contact and touching, rooming in so there is no unnecessary separation and teaching mothers to tune into their newborn’s cues and respond appropriately to enhance maternal synchrony. Additional features may be added based upon your phase 1 findings. During your scoping review watch out for programs like ‘tuned-in parenting’ offered through Ngala in Western Australia, Circle of Security International [relationship based

early intervention program designed to enhance attachment security between parents and children] and Mother-Baby Nurture groups in WA. You may have to contact people from their websites to find information about these programs if publications are not readily available. Will training/education be available to all midwives working on the postpartum ward or just midwives working in an MGP model who provide care up to 6 weeks post birth?

As discussed for **phase 2**, a test-retest process to determine reliability and a Cronbach alpha score requires a larger sample size than 5 or 8. I have never seen numbers smaller than 50 for each test so would suggest you consult a statistician for advice. Generally this measure of scale reliability is employed for an instrument that is a scale (where items are added together to generate a total score such as the EPDS). Something for consideration: There are existing validated and reliable satisfaction instruments and mother-infant attachment instruments that could be modified rather than developing new surveys. You can then run a Cronbach Alpha after phase three to confirm that your modifications haven't jeopardised the reliability (Cronbach Alpha remains > 0.7).

In the description in the methods of **phase 3**, it appears that you may be conducting a before/after research design? When will women be recruited for phase 3? During late pregnancy? Exactly when will women be asked to complete the 'before' data using the postnatal bonding scale and maternal infant responsiveness scale (assuming they self-administered)? How can women complete the surveys 'before' the intervention when midwives will be supporting the mothers from birth onward and ideally implementing the intervention immediately post birth starting with skin to skin contact? Is it feasible to collect 'before' data toward the end of pregnancy (unable to assess without seeing the surveys)? Will only women who are being cared for by the 10 midwives recruited and trained in implementing the intervention (noted on page 21) be invited to participate in this phase? When and how will the 'after' measurements be collected (6 weeks post birth) via a postal or online survey?

Have you considered a pilot cluster RCT where women from one institution receive the intervention from trained midwives working in a MGP model and a comparison group from another institution receive standard care and collect bonding and maternal infant responsiveness data from both groups at 6 weeks post birth?

Are there any cost implications for this proposed study?	Yes ☒	No ☐
If yes, have these costs been addressed in the proposal?	Yes ☒	No ☐

Please include additional typed information, comments and critique of the proposal that can be sent to the candidate and supervisors

Reviewer signature: ___ Yvonne Hauck ____ Date: _____October 1, 2020_____
